# Supplementary material for: Historic and recent trends in county-level coronary heart disease death rates by race, gender, and age group, United States, 1979-2017
Source: PLoS One. 2020 Jul 7;15(7):e0235839. doi: 10.1371/journal.pone.0235839 (PMC7340306; doi:10.1371/journal.pone.0235839)
Supplement: Attachment — Submitted filename: Response to Reviewers.docx [file pone.0235839.s002.docx]

Thank you for the thorough review of this paper. Our responses to each comment are presented in *italics* below.

Reviewer #1: The following reflects a biostatistical review of the manuscript.

In this work, the authors perform a thorough examination of geographic variation in coronary heart disease (CHD) mortality, and in temporal trends for CHD mortality. They used a Bayesian multivariate space-time autoregressive model, allowing for "borrowing strength" in estimation, for neighboring counties, years, and demographic groups. Specifically, a strength of this method is that it allows for stable estimation of rates and trends even in years/ages/counties/demographic groups with very few observations. The authors identified significant spatiotemporal variation in CHD rates. In particular, they identified counties with recent increases in CHD mortality, going against the overall national trend of CHD mortality reduction.

While this manuscript is excellent overall, I believe it would benefit from some relatively minor changes. In particular, since the modeling is such a critical part of the work, it would greatly strengthen the manuscript to include a more detailed description of the model details. The results are difficult to interpret without the context of the model details, and it is appropriate to include those details within this manuscript rather than requiring the reader to look elsewhere.

*Response: We have modified the methods (page 5) to include much greater detail about the model.*

The methods section (line 71) states that the model is able to borrow strength across adjacent demographic groups. How is adjacency defined in this context? Aside from adjacency between age groups, adjacency relationships (and the strength of these relationships) between demographic groups are not obvious. Please include details on this in the methods section.

*Response: We have included these details in the methods (page 5).*

How were the four intervals (1979-1990, 1990-2000, 2000-2010, 2010-2017) chosen for the APC calculations?

*Response: We have included a description of how those intervals were chosen in the methods (line 108).*

Reviewer #2: Vaughan et al examined the trends of county-level death rates due to coronary heart disease in the US from 1797 to 2017. My comments are listed below:
1. Line 65: in this section it is not clear whether the death rates were crude rates or standardized/adjusted rates. From the tables presented it seems that the rates were age standardized. If this is the case, please specify it in the methods.

*Response: All death rates were age-standardized using the 2000 US standard population. This was previously included in the manuscript (line 83), so no change has been made.*

2. Line 77: Please provide rationale for only including death at age 35 and above? And the reason of categorizing age groups as 35-64, ages 65 and older?

*Response: Deaths were only included for ages 35 and older because deaths from CHD below age 35 are exceedingly rare. Including these deaths would therefore add to the denominator but not the numerator, lowering death rates in this middle-aged group. We selected these two age groups based on both past work and on public health programs. Our prior work found that ages 35-64 have experienced widespread increases in death rates due to cardiovascular disease, but these death rates have continued to decline for ages 65 and older. Additionally, national initiatives such as Million Hearts include adults ages 35-64 as a target population. Our inclusion of this age group in this paper therefore directly informs public health practice. We have clarified this in the introduction (line 47) and in the methods (line 70).*

3. Line 233-238: The authors should consider the possibility of changes in the validity of death certificate coding during the study period. With greater focus on population levels of blood pressure its plausible that the validity of codes assigning cause of death to coronary heart disease may have not been stable over this time period. And how this can affect the results.

*Response: We have included this as a limitation (line 263).*

Reviewer #3: This is a well-written, important manuscript that provides key data on changes in CHD mortality rates in the US over time. There are additional analyses/considerations that could strengthen the findings of this paper.
1. In the introduction, there is no acknowledgement of work on premature CVD mortality as recently published by Chen and colleagues (JAMA Cardiology doi:10.1001/jamacardio.2019.3891). This work should be cited and briefly described.

*Response: Thank you for pointing out this key reference. We have included this reference and its context in the introduction (line 42).*

2. Why were those aged 35-64 combined into one group? CHD events before age 50 may be different from those after age 50 (could be more related to genetic susceptibility). Were there considerations about dividing up the groups 35-49 vs. 50-64 vs. 65+?

*Response: Deaths were only included for ages 35 and older because deaths from CHD below age 35 are exceedingly rare. Including these deaths would therefore add to the denominator but not the numerator, lowering death rates in this middle-aged group. We selected these two age groups based on both past work and on public health programs. Our prior work found that ages 35-64 have experienced widespread increases in death rates due to cardiovascular disease, but these death rates have continued to decline for ages 65 and older. Additionally, national initiatives such as Million Hearts include adults ages 35-64 as a target population. Our inclusion of this age group in this paper therefore directly informs public health practice. We have clarified this in the introduction (line 47) and in the methods (line 70).*
